# An assessment of the potential environmental effects of bridge construction in Boga, Patuakhali, Bangladesh

**DOI:** 10.1016/j.heliyon.2023.e16562

**Published:** 2023-05-23

**Authors:** Md. Tariqul Islam, Mawya Siddeqa, Ananya Mukherjee, Shakina Afroz Bithi, Songita Mandal, Maksudul Islam

**Affiliations:** a Department of Environmental Science, Patuakhali Science and Technology University, Dumki, Patuakhali 8602, Bangladesh; b Department of Geo-Information Science and Earth Observation, Patuakhali Science and Technology University, Dumki, Patuakhali 8602, Bangladesh; c Institute of Disaster Management, Khulna University of Engineering & Technology, Khulna 9203, Bangladesh

**Keywords:** Boga Bridge, Environmental impact assessment (EIA), Environmental consequences, Leopold matrix, Mitigation measures

## Abstract

Bangladesh Road Transport Authority announced a plan to build a bridge over the Lohalia River in Boga, Patuakhali, which will significantly modify the entire communication system and lead to significant economic empowerment in the southeastern part of Bangladesh. This study was designed to help decision-makers through the identification and evaluation of all potential social and environmental consequences of this proposed project using an integrated methodology combining GIS mapping, environmental impact value assessment, and critical evaluation of the environmental impact through the Leopold matrix. The necessary information required for this study has been collected through questionnaire surveys, participatory community risk assessments (CRA), focused group discussions, key informant interviews, and reviews of previously published documents. According to this study, the proposed Boga Bridge construction will have some adverse environmental consequences including agricultural land and productivity loss, the decline of ecosystem health, extinction of endangered species, deterioration of water, air and soil quality, sedimentation and changes in river flow. Despite these adverse impacts this project will improve the life and livelihood of the coastal community and foster economic growth and industrialization over the long run through easily assessable road transportation. Additionally, the estimated overall environmental impact value (−2) and Leopold matrix's impact value (−1.51) revealed that this project has low adverse effects on the surrounding environment. Moreover, the majority of the environmental consequences were found to be transient because they were only limited to the construction phase which makes it simple to control with the proper implementation of appropriate mitigation strategies. Therefore, this study furnished some effective mitigation strategies incorporating mitigation hierarchy principals to avoid and minimize adverse impacts as well as enhance the positive impacts of this project. Finally, this study recommends constructing the proposed Boga Bridge after ensuring rigorous implementation and monitoring of all impact mitigation strategies proposed in this study.

## 1 Introduction

The southern coastal region of Bangladesh is getting more economic importance from the government and hence going through a revolutionary development in the infrastructure sector. The coastal rural highway network in Bangladesh is crucial for enhancing the health, education, and livelihoods of roughly 66% of the country's population living in the coastal area because economic development and social cohesion greatly depend on communication facilities [1,2]. Since the completion of the Padma and Payra bridges, the economic empowerment and communication infrastructure of the coastal region have changed [[3], [4], [5], [6]]. But the people of Bauphal, Dashmina, and Galachipa upazilas are not getting the actual benefit of Padma and Payra bridges as they till now have to rely on the ferry system for crossing the Lohalia River [1,7,8].

The Lohalia River, which separates Bauphal and Dumki upazila is one of the biggest rivers in the Patuakhali district and is tidally impacted, meandering, and dynamic [1,8]. This river is the main transport barrier between the Bauphal, Dashmina, Galachipa, and Patuakhali [8]. People must wait for an average of one and a half hours or longer to cross the river because there is only a ferry service that provides connectivity with other locations. Road connectivity is greatly hampered at these points by ferry crossing, especially during the flood season [[7], [8], [9]]. Moreover, this ferry service is seriously interrupted during natural disasters, which significantly impairs economic and social activity [4]. About 2 million residents of the southern upazilas of Bauphal, Dasmina, and Galachipa have been calling for the construction of a bridge at Boga Ferry Ghat in Bauphal for a long time to speed up communication by road to Dumki, Patuakhali, Barishal, and the capital. All three upazilas would be connected to Dumki, Patuakhali, Barishal, and the rest of the nation if a bridge was constructed at this location, and the irony of the ferry crossing in the way would no longer exist in the connection [1,7,8,10].

The government of Bangladesh has decided to build a bridge there after learning about the challenges faced by residents of these three coastal upazilas due to the lack of a bridge over the Lohalia River. A high-level team of the government consulted with China about the feasibility and overall cost of the bridge construction. After that, on May 11, 2017, the Chinese Ambassador and the Secretary of the Economic Relations Division (ERD) in the 10.13039/501100005045Ministry of Finance of Bangladesh signed a grant agreement worth RMB 500 million for the construction of a 400 m long bridge over the Lohalia River at the Boga point on the Lebukhali-Dumki-Bauphal-Dashmina-Galachipa road [10,11]. The southern region's economy, in particular Boga, Bauphal, and Kalaiya, will soon flourish after the bridge is finished [7,8,12,13]. The construction of this bridge will result in time and cost savings for the transportation of people and commodities and will make it simple to carry agricultural products generated in three upazilas to other parts of the nation, including the capital [1,7,8,14,15].

Based on location, magnitude, and the degree of potential pollution, the Environmental Conservation Rules (1997) divided all development projects and industries into four categories: green, orange A, orange B, and red, with correspondingly, no, minor, medium, and severe environmental impacts [[16], [17], [18]]. This classification states that the initial environmental examination (IEE) and EIA are not necessary for projects in the green category. On the other hand, red-category projects demand IEE and EIA before the start of the construction process [16,18]. This classification places all regional and national roadway, railway, and bridge projects that are longer than 100 m in the “red” category [8,16,19]. As the proposed Boga Bridge is 400 *m* in length [10], it is generally included in the red category and must have some moderate to severe possible significant negative impacts on the environmental parameters. Therefore, according to the Environmental Conservation Rules (1997), it was necessary to undertake an EIA before beginning the planning and construction work on the Boga Bridge project to identify any potential negative effects and potential effective impact mitigation strategies [8,16,18].

Environmental Impact Assessment (EIA) is a systematic and comprehensive procedure for evaluating potential effects before deciding whether or not to approve a plan to proceed [[20], [21], [22]]. A long time ago, when the initial planning of this project was underway, Islam et al. and Hasan et al. conducted their investigation to identify the potential environmental implications of the Boga Bridge project [1,8]. According to their study, this project may have some significant adverse environmental effects, such as the loss of agricultural land and livelihood opportunities, the decline of fisheries and aquatic habitat, the extinction of native tree species, the alteration of the river's course, the growth of char land, navigational issues, changes to the river's flow, surface water pollution, air pollution, soil contamination, accidents, and so on [1,7,8,14]. However, there was no evidence that this project has progressed to the building stage after five years since planning and budget allocation [10]. The initial environmental conditions of the project's surrounding area may have altered during this extended period. There is also a possibility that the overall effects of this project on the nearby environmental components that were discovered in the early research conducted by Islam et al. and Hasan et al. may change if these initial environmental changes take place [1,8].

Recently, feasibility studies and other necessary activities for the construction of the Boga Bridge have already been started by roads and highway departments. This project is now in the tendering process and the construction work will be started after the completion of the Bekutia Bridge over the Kacha River at Pirojpur [23]. Therefore, this study was designed to assess and evaluate all the significant environmental effects of this project at each of its construction, pre-construction, and operational phases using an integrated analytical process to aid the decision-making process. This study applied an integration of GIS assessment, environmental impact value calculation and Leopold matrix-based critical evaluation of impacts for the identification and evaluation of all potential environmental impacts of this proposed bridge. Additionally, this study used the analytical hierarchy process (AHP) to perform a multi-criteria decision analysis and involved a wide range of stakeholders in the decision-making process after informing them of all the estimated social and environmental effects of the proposed project. Last but not least, based on the stakeholders' decisions analysis and discovered adverse consequences in this study, we explored effective impact mitigation strategies against all of the reported negative impacts employing the principles of mitigation hierarchy [24]. This study was unique since it was the first of its kind to use an integrated multidimensional methodology and to involve a wide range of stakeholders in both the assessment and evaluation of the project's potential environmental effects and the decision-making process. The integrated approach adopted in this study offers a larger perspective on the interactions between various project activities and environmental components as well as the significance of these interactions which combinedly help us to better comprehend the project's overall environmental implications. Finally, by going through this integrated process, we were able to create more effective and comprehensive impact mitigation strategies for this project that consider all of its significant environmental effects.

## 2 Materials and methods

### 2.1 Project location and access way

**Fig. 1:**
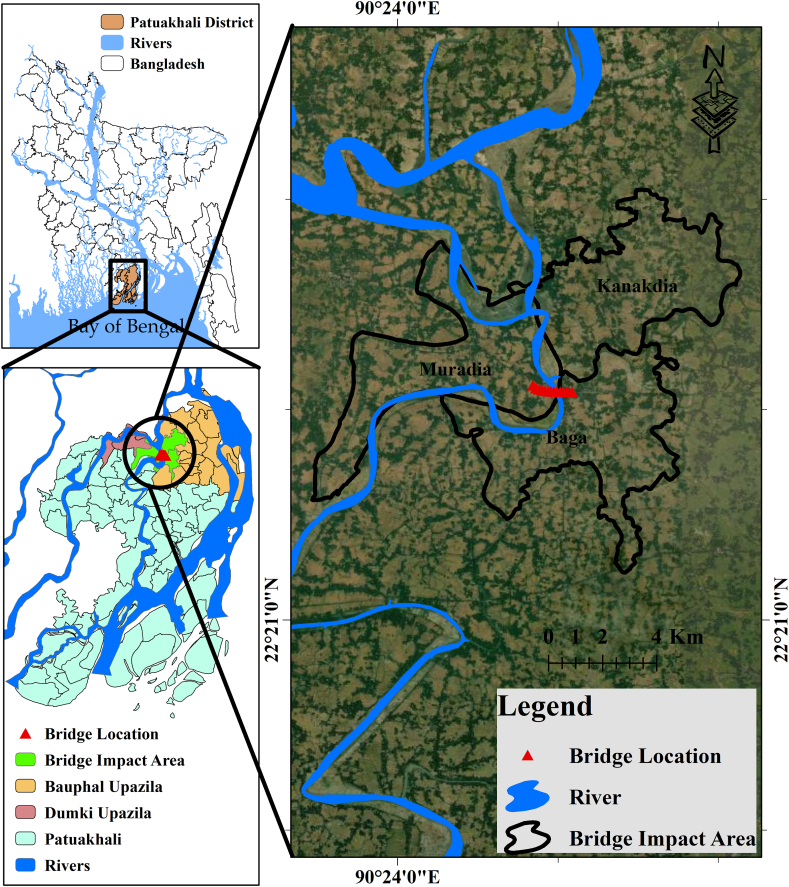
Map showing the proposed bridge location and surrounding area.

The proposed 9^th^ Bangladesh-China Friendship Bridge popularly known as the Boga Bridge (also written Baga) project will be built over the Lohalia River. The proposed project is situated in the southern part of the country, in the Boga ferry Ghat of Bauphal Upazila, on an area of land that is 7041 acres large. The project's geographical coordinates are 22°42′0″ North latitude and 90°45′0″ East longitude (Fig. 1). The proposed project site is easily accessible by both roads and riverways and is situated at a distance of 28.8 km from Pauakhali Sadar & 294 km from Dhaka by roadways and 15 & 200 km from Patuakhali Sadar & Dhaka respectively by waterways [25].

### 2.2 Data collection

The survey approach is crucial for obtaining correct data [8,26]. In order to observe the project location and the local environmental conditions, the study region was first visited. A combination of primary and secondary sources was used to gather the data. Primary data was gathered via questionnaire surveys, focused group discussions (FGDs), key informants’ interviews (KIIs), participatory community risk assessments (CRAs), field observation, and other appropriate methods. Local experts and knowledgeable persons, including political leaders, teachers, traders, and community members were personally surveyed to know their perceptions about the environmental and socio-economic impacts of the proposed project. Additionally, they were asked to suggest any potential solutions the project may use based on their knowledge and experiences. Through literature reviews, publications, journal articles, field studies, and meetings with various stakeholders, secondary data was gathered.

### 2.3 Geo-spatial analysis of bridge surrounding environmental and physiochemical parameters

This study conducted a GIS and RS-based geospatial analysis and mapping of different environmental and physiochemical components to generate a wider view of the bridge surroundings. Landsat 9 OLI/TIRS, NASA 30 *m* SRTM DEM, ISRIC 250 *m* world soil information, waterbody and roads shapefile, and world's time series precipitation data 2011–2020 datasets were used in this study for geospatial analysis [[27], [28], [29], [30], [31]]. The land use and land cover (LULC) map was created by the supervised classification of the Landsat image. NDVI map was prepared using the ratio between the visible red (R) and near-infrared (NIR) bands (eq. (1)) where band 4 and band 5 represented R and NIR Respectively.

(1) $$NDVI=\frac{\left(NIR-R\right)}{\left(NIR+R\right)}$$

Distance from water and distance from roads maps were prepared using Euclidian distance techniques under the hydrology tools from ArcGIS's spatial analyst tools. A drainage density map was prepared from the SRTM 30 *m* global DEM data and a slope map was also prepared from here using slope creation tools. Soil type and precipitation maps were prepared by digitizing and processing ISRIC's 250 *m* soil texture data and Climatic Research Unit's world precipitation data respectively.

### 2.4 Environmental impact value assessment

Various ecological, physiochemical, sociocultural, and human-interest parameters were used in this study to estimate the environmental impact value (EIV), and the potential environmental consequences were also determined under those parameters [8,32]. The qualitative assessment of the degree of impact was followed by a calculation of the project's total potential environmental effects, both inside and beyond the project area. The degree of impact value was assigned according to the findings of different impact assessment studies for similar bridge related large projects in Bangladesh [7,14]. In this study, the impacts of this project on different environmental parameters were evaluated by assigning a score ranging from 0 to ±5 for both positive (+) and negative (−) impacts. Changes in environmental parameters consider Severe (+5 or −5), Higher (+4 or −4), Moderate (+3 or −3), Low (+2 or −2), Very Low (+1 or −1) and No change (0) [32]. The environmental impact value (EIV) for this proposed project was estimated using eq. (2) mentioned below [14,[32], [33], [34]]:

(2) $$EIV=\sum_{i=1}^{n}\left(Vi\right)Wi$$

where, EIV = Environmental Impact Value, Vi = Relative change of the environmental quality of parameters, Wi = Relative importance or weight or parameter, N = Total number of environmental parameters.

### 2.5 Environmental impact evaluation using Leopold matrix

The Leopold matrix analysis is a tool developed by L.B. Leopold which is widely used for the identification and evaluation of potential environmental effects of any proposed project to systematically measure the overall effectiveness of that project [[35], [36], [37]]. This study adapted the Leopold matrix to critically evaluate all the potential environmental effects of this project along with their significance to aiding in decision-making and the creation of appropriate impact mitigation strategies. There used 16 impact factors mainly representing bridge construction and operation activities in the horizontal axis which may have potential impacts on the selected 17 environmental and social components placed in the vertical axis of the matrix [37,38]. After that the cells where any potential impacts may occur were identified critically and a diagonal line was drawn from the lower left corner to the upper right corner in that cell to place the impact magnitude and importance of that impact. The magnitude of all the possible impact were then placed in the upper left-hand corner of the diagonal line and the importance of that respective impact was in the lower right-hand corner of the diagonal line for each impact cell [39]. Although the original Leopold matrix used a scale of 1–10 to weigh magnitude and importance, this study used a modified scale of 1–5, with 1 denoting very low magnitude and importance and 5 denoting very high magnitude and importance [35,36,40]. In case of negative impact, this study used a (−) sign before the score for weighting the magnitude of that adverse impact [39]. The magnitude was scored based on the severity and extent of the impact and the importance of that impact was scored based on the significance of the impact. After the scoring process was complete, the magnitude (M) and importance (I) values of each impact cell were multiplied to estimate the significance (s) of that cell and highly significant environmental impacts were then identified using eq. (3). Later, the significance of all impact cells was summed to get the overall significance of the project activities (eq. (4)), and the overall project impacts were estimated from eq. (5) [39]. Finally, required impact mitigation measures were developed on a prioritized basis depending on the identified most significant environmental impacts and the overall project impact value.

(3) ImpactSignificance(s)=(M)×(I)

(4) OverallSignificance(S)=∑{(M)×(I)}

(5) $$Project\;Impact\;Value\;\left(PIV\right)=\frac{\sum \left\{\left(M\right)\times \left(I\right)\right\}}{\sum I}$$

### 2.6 Multi-criteria decision analysis with analytical hierarchy process (AHP)

After conducting an integrated assessment of all potential environmental effects of the Boga Bridge, we further involve a wide range of stakeholders, including knowledgeable locals and government officials to formulate an appropriate decision hierarchy with their priority rank for eradicating all adverse effects [41]. Analytical Hierarchy Process (AHP) based Multi-criteria Decision Analysis (MCDA) was used to perform this work in a participatory approach. In order to make an acceptable conclusion about the construction of the Boga Bridge, nine alternative decision-supporting options were first formulated through expert discussions, and those criteria were then further assessed through the AHP (Table 1).

**Table 1:** Selected decision-supporting options for the pairwise comparison process.

Option number	Decision supportive options
1	There required no mitigation measures for this bridge construction
2	Selection of alternative site for bridge construction
3	Modification of bridge design and materials for reducing adverse impacts
4	The bridge should be constructed with effective mitigation measures
5	Robust monitoring and evaluation of mitigation measures to ensure their effectiveness
6	Capacitate and engage the local community in the post-construction management
7	Early engagement of stakeholders and local indigenous peoples in project design for the effectiveness of bridge
8	Improve existing ferry facility rather than bridge construction
9	This bridge shouldn't be constructed as it has several negative impacts

After that, this study prepared a checklist consisting of a total of 36 pairwise comparisons with selected 9 options and collected responses from the selected stakeholders for the priority ranking of appropriate decisions (Table S1). In pairwise comparisons, the relative importance of each criterion was determined using Saaty's AHP scale, where 1 indicates the equal importance of two criteria and 9 indicates the extreme importance of one criterion over another [42]. A square matrix was generated with the relative importance weight of all criteria for the pairwise comparisons of each participant. The row of the matrix represents each choice criterion, and the column shows the link between all of these criteria. After that, a consolidated weighted matrix was created from these response matrices using the geometric mean of all the matrices. The priority ranks of all decision criteria were finally estimated from the calculated normalized principal eigenvector or priority vector. All criteria were then normalized to a scale of 0–1, with 1 denoting the highest importance and 0 denoting the lowest importance, to calculate the priority vector from the weighted matrix. For the normalization, the matrix's sum for each column was first determined, and all the criteria for each column were then divided by that sum. Following that, each row's sum was calculated from the normalized matrix to obtain the priority value, and each calculated value was then further normalized to obtain the priority vector and priority rank [43].

## 3 Results

### 3.1 Project surrounding environmental & physiochemical components

**Fig. 2:**
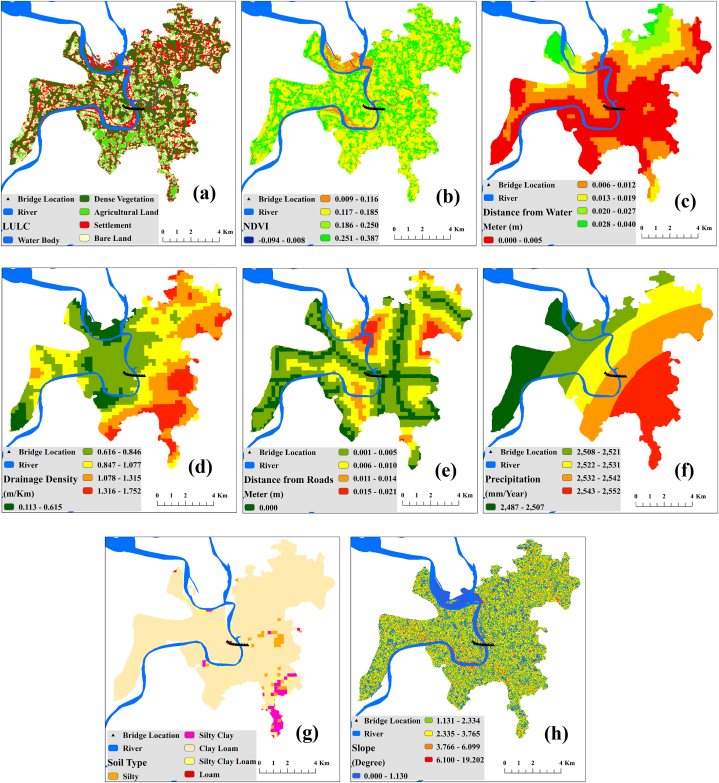
Geo-spatial maps of bridge surroundings environmental and physiochemical components (a) land use and land cover (LULC); (b) NDVI; (c) distance from water; (d) drainage density; (e) distance from roads; (f) precipitation; (g) soil type; (h) slope.

GIS and RS-based geospatial analysis of different environmental and physiochemical components (Fig. 2) provides a greater understanding of the project surrounding immediate environment and helped us to anticipate possible environmental consequences for this proposed project. This analysis showed that the western part of the proposed bridge and approach road passed through the agricultural lands and the eastern part passed through the dense vegetation area which indicates a lot of green vegetation and agricultural lands will be damaged by this project (Fig. 2a and b). Additionally, there are areas of moderate settlement at both ends of the bridge. The bridge has strong and well-developed road connectivity with all the upazila situated at the eastern end (Fig. 2e). The proximity of this project to water bodies including rivers, canals, streams, and ponds indicates that they are all nearby (Fig. 2c). However, the area upstream of the bridge has a moderate to high drainage density, and a significant amount of water flows through this section of the river (Fig. 2d). The proposed project will be built in a flat location with clay loam and silty clay loam soil that receives moderate to heavy precipitation annually (Fig. 2h, g, f).

### 3.2 Significant impacts of the proposed project

The proposed Boga Bridge construction project will affect the environment in both favorable and unfavorable ways during all of its construction, pre-construction, and post-operation phases. The significant environmental consequences of this project are described here based on people's perceptions, field observations, and geospatial analysis.

A. Negative impacts
  i. **Loss of agricultural land and resettlement:** A total of 3.04 sq. km of land will be needed for the implementation of this project of which 1.58 sq. km would be used to build the approach road. In addition, a total of 65 households with 212 people will need to relocate for this land acquisition [9]. These displaced people belong to the landless, poor, and vulnerable groups and they will have to forcedly adjust to the new settlement place which may have a great negative impact on their income, mind, and culture.
  ii. **Development of resettlement sites:** For the resettlement of the displaced person, a resettlement site will be constructed which will require 0.12 sq. km of land [9]. Wetlands and some agricultural land will be destroyed during this land acquisition, which could have an impact on the livelihood of people.
  iii. **Loss of aquatic habitat:** The project might have some major impacts on the habitat of aquatic life, some of them are temporary and some of them are permanent. The Lohalia River, floodplains, and ponds are the three types of aquatic habitats that surround the planned bridge. The aquatic environment will be destroyed when the soft sediment of the river bed will be replaced with hard concrete during the construction of various bridge structures, approach roads, and river training works.
  iv. **Loss of vegetation and trees:** A significant number of trees like bananas, bamboo, papaya, firewood, medicinal trees, and other household trees will be destroyed when some project lands will be acquired from the household area. Additionally, cutting a significant number of trees from acquired land and for site preparation will have a significant negative impact on the environment.
  v. **Loss of agricultural production:** A total of 3.16 sq. km of land will be permanently required for this project and most of this land is usually used for agricultural production which will approximately reduce a total of 1187 tons of crop production in this area. The main crops grown here are rice, wheat, onions, garlic, and potatoes, and land acquisition will have the biggest impact on the production of rice and potato [2].
  vi. **Impact on endangered species:** Different fishes, turtles, and terrestrial birds are the endangered species found in Boga Rivers which might have potentially major impacts from this project. They are likely to avoid the construction area due to high noise levels which may also impact their behaviors and breeding pattern. Additionally, as some ponds and flood plains will be filled during the construction phase, a huge number of fish and some birds amphibians, and reptile species will be lost from the area.
  vii. **Impact on surface water quality:** The quality of the river's water will decline due to the construction of the bridge superstructure, river training projects, and other components. In addition, the site's development, maintenance, and decommissioning activities may cause fuel leaks and oil spills that will contaminate nearby bodies of water. The dumping of various wastes will lessen the turbidity of the water downstream, which could have a major negative impact on fish breeding and spawning as well as affect aquatic biodiversity.
  viii. **Noise pollution:** Various construction activities, such as casting, welding, and transporting materials will produce severe noise and most of the time noise levels will exceed acceptable standards. Additionally, during the operational phase, a great deal of noise will be produced by the massive transportation of various motor vehicles over the bridge. Even though the neighborhood is far from the construction site, the workers there will be directly impacted [44].
  ix. **Impact on air quality:** Construction activity will generate different types of dust and particulate matter which will deteriorate surrounding air quality. Different types of wastewater, solid waste, and organic waste dumped from the construction camp will spread foul odors and contaminate the local air quality. Additionally, the high volume of motor vehicle traffic over the main bridge during operation will release a lot of gases, such as CO, CO_2_, SO_2_, etc., which will eventually contaminate the air.
  x. **Accident and health risks:** There is a possibility of an increased number of accidents during different construction activities as well as bridge operational phases. There is also an increased safety risk for women and children, and the possible increase of infectious diseases while living in the construction camps. Moreover, improper sanitation facilities can increase waterborne diseases.
  xi. **Loss of income and livelihood:** Some people will suffer long-term consequences as a result of the project's implementation because they will lose their livelihood and stable source of income. Though some people will receive temporary employment chances throughout the project, they may afterward experience problems with unemployment.
B. Positive impacts
  i. **Easy transportation:** The ferry barrier between the Bauphal, Dashmina, Galachipa, and Dumki, Patuakhali will be eliminated upon completion of the bridge. Moreover, this bridge will link this coastal region with the rest of the nation, allowing for simple movement and a quick communication system.
  ii. **Generation of employment:** The proposed bridge construction project will generate new employment opportunities for locals. During different construction activities, about 220 skilled, 450 unskilled, and 7 experts will get short-time employment opportunities in this project.
  iii. **Gender promotion:** During road construction, a large number of female laborers and experts will get employment opportunities. Since female laborers are more readily available and are generally less expensive, contractors will employ more women in different stages of the construction.
  iv. **Roadside vegetation:** There will be extensive tree plantation around the open place of the bridge and both sides of the approach road. In the long term, this improved landscape and green environment will bring comfort and a pleasing appearance to the locality.
  v. **Land use change:** Long-term effects of the bridge include a progressive change in the land use pattern of the project region and its surroundings toward industrialization and urbanization.
  vi. **Economic development:** When this bridge will link coastal Bauphal, Dashmina, and Galachipa upazilas to Dumki, Patuakhali, Barishal, and the rest of the world, it will not only facilitate transportation and movement but as a whole, it will bring significant economic growth and social benefits.

### 3.3 Environmental impact value estimation

This study estimated the overall environmental impact value for the construction of the proposed Boga bridge by dividing all the parameters into ecological, physiochemical, sociocultural, and human-interest parameters using equation (2), and the results are shown in Table 2. The overall EIV of the Boga bridge project was computed by combining all the calculated values for all categories after obtaining category-wise individual EIV.

**Table 2:** Estimation of environmental impact value (EIV).

Parameters category	Parameters	Relative change (Vi)	Relative importance (Wi)	Individual EIV (Vi*Wi)	EIV
Ecological	Fisheries	30	−2	−60	−166
	Ecosystem	17	−2	−34	
	Endangered species	7	−3	−21	
	Flora and Fauna diversity	9	−2	−18	
	Loss of vegetation	13	−3	−39	
	Wetland	13	0	0	
	Char land	13	−2	−26	
	Plantation	16	+2	32	
Physio-chemical	Water	12	−2	−24	−61
	Air	8	−2	−16	
	Noise	15	−1	−15	
	Vibration	12	−1	−12	
	Topsoil	11	−2	−22	
	Surface water quality	5	−1	−5	
	Waste generation	12	−3	−36	
	Change river flow	13	−2	−30	
	Water logging	10	0	0	
	Economic development	22	+3	+66	
	Reuse of topsoil	17	+1	+17	
	River training works	16	+1	+16	
Socio-cultural	Livelihood and income	18	−3	−54	−33
	Safety and health risks	9	−2	−18	
	Occurrence of accident	13	+3	+39	
Human-interest	Agricultural lands and production	14	−3	−42	+258
	Self- relocation	14	−3	−42	
	Employment generation	20	+3	+60	
	Commercial and service facilities	16	+2	+32	
	Gender promotion	13	+2	+26	
	Change in land use	4	+2	+8	
	Road communication	30	+5	+150	
	Flood protection	5	+1	+5	
	Migration	7	+2	+14	
	Education	8	+2	+16	
	Aesthetic value and recreation	5	+1	+5	
	Community upgradation	13	+2	+26	
**Overall Project EIV**					**−2**

From Table 2, the overall Environmental Impact Value (EIV) for ecological parameters was found “-166” which indicates this project might have severe negative impacts on the surrounding ecological parameters. Table 2 also indicates that fisheries, vegetation, ecosystem, and char land subcategories were affected most by this project. The calculated EIV “-61” indicates a moderate negative impact on the physiochemical parameters where waste generation, river flow change, water quality, and topsoil erosion were the most vulnerable impacts of this project. Similarly, this project also has moderate negative impacts on the socio-cultural parameters as the estimated EIV was “-33” for this category. The negative effects in this category included fewer opportunities for livelihood and income as well as increased risks to safety and health. On the other side, this study also discovered several positive human-interest variables that are supportive of the construction of the Boga Bridge. The calculated overall EIV for the human-interest parameters was "+258,” indicating that the general public has a favorable perception of the project's construction. The public's positive opinions of this project are most strongly influenced by the enhancement of road communication, which is followed by economic growth, employment creation, enhanced commercial service facilities, gender promotion, community upgradation, and a decline in accidents. The final result of our estimated overall EIV was "-2,” indicating that the project's construction activities had a minimally detrimental effect on various environmental factors.

### 3.4 Critical evaluation of environmental impacts

Leopold matrix presented in Table 3 has demonstrated the effects of the proposed Boga Bridge project's activities on various environmental factors at various stages of construction. The proposed project activities have a low to moderate negative influence on various environmental components around the project, according to the estimated total project impact value (PIV) of impact factors on different environmental components, which was found to be “-1.51”. Several physiochemical and ecological components including water quality, air quality, topsoil and cropland, ecosystem, endangered species, flora & fauna diversity, wetland & fisheries, and char lands would face adverse impacts through different activities during pre-construction and construction phases. Likewise, pre-construction and construction operations including land acquisition, site cleaning, construction yards and camp, and waste dumping will have minimal negative effects on the sociocultural and human-interest dimension, which includes land usage, employment, and accident and health risk. Water and air quality, topsoil & cropland, ecosystem, flora & fauna diversity, and wetlands & fisheries would be the most affected environmental components for this project's construction activities. Despite these unfavorable effects, the bridge would have positive effects on the economy, employment, community improvement, land use patterns, and communication system, which would increase the project's overall effectiveness.

**Table 3:**
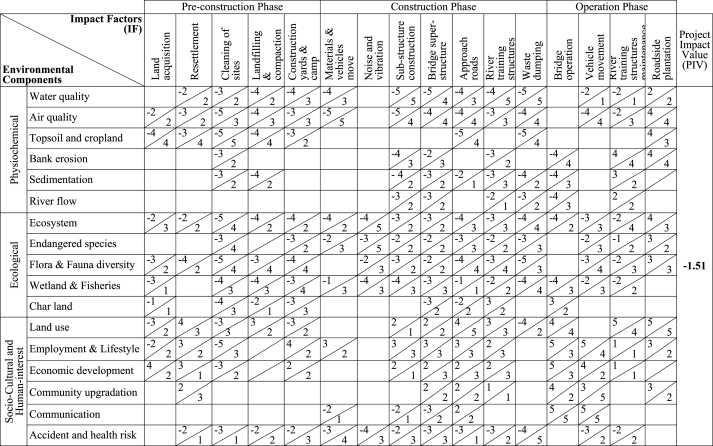
Magnitude and impact matrix of the Boga Bridge construction project.

### 3.5 Multi-criteria decision analysis

A larger and more detailed perspective on the stakeholders' perceptions of the proposed Boga Bridge was revealed using multi-criteria decision analysis with an analytical hierarchy process (Table 4). As this decision analysis was carried out following the conclusion of all impact assessment steps, it aided in understanding the best course of action for the construction of the Boga Bridge. Though this proposed project has low to moderate overall environmental consequences and has significant adverse effects in its construction period, the majority of respondents preferred to build the bridge using the existing design and the proposed location incorporating appropriate mitigation measures. Additionally, a robust monitoring and evaluation of the implemented mitigation measures is also required for the effectiveness of this bridge according to the respondents. Furthermore, they emphasize capacitating and engaging stakeholders and local indigenous peoples in the project design and post-construction management to improve bridge effectiveness and lessen environmental consequences. On the other hand, some respondents preferred choosing a different bridge location and altering the current bridge design to prevent negative environmental effects.

**Table 4:** Decision matrix and priority rank of decision options.

Matrix	Option 1	Option 2	Option 3	Option 4	Option 5	Option 6	Option 7	Option 8	Option 9	Normalized Priority Vector	Priority (%)	Rank
Option 1	1	1/5	1/6	1/7	1/6	1/4	1/4	2	4	0.0356	3.56	7
Option 2	5	1	1	1/5	1/3	1/2	1/2	5	6	0.0850	8.50	6
Option 3	6	1	1	1/5	1/3	1/2	1/2	5	6	0.0883	8.83	5
Option 4	7	5	5	1	4	5	6	8	9	0.3600	36	1
Option 5	6	3	3	1/4	1	3	2	6	7	0.1754	17.54	2
Option 6	4	2	2	1/5	1/3	1	1	5	6	0.1065	10.65	4
Option 7	4	2	2	1/6	1/2	1	1	5	6	0.1078	10.78	3
Option 8	1/2	1/5	1/5	1/8	1/6	1/5	1/5	1	1	0.0222	2.22	8
Option 9	1/4	1/6	1/6	1/9	1/7	1/6	1/6	1	1	0.0194	1.94	9

### 3.6 Environmental impact mitigation measures

**Fig. 3:**
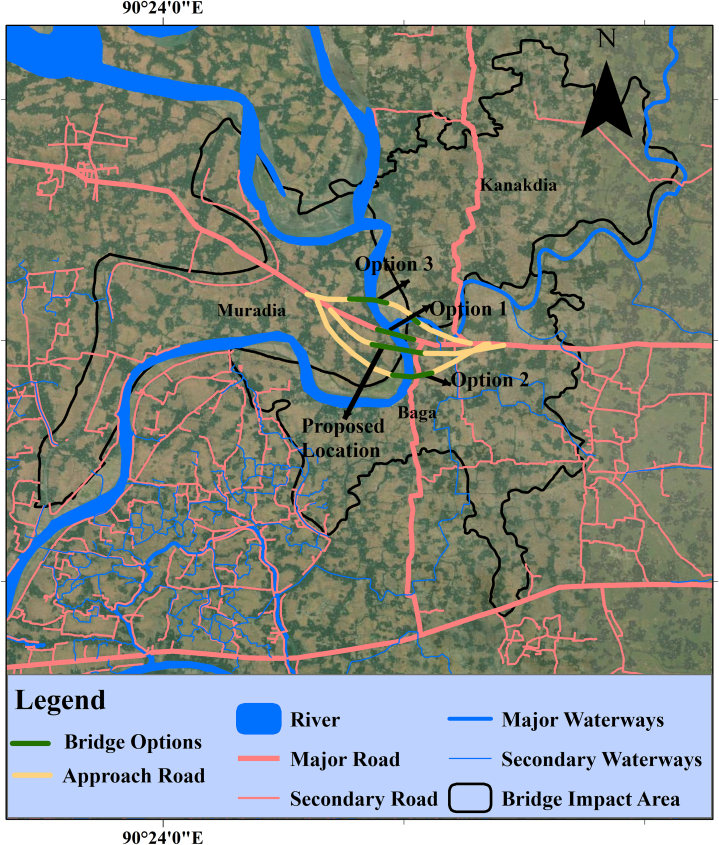
Bridge alternative location analysis.

Any development project's environmental sustainability can be ensured by using a proper environmental management strategy and mitigating strategies. According to our analysis, the proposed Boga Bridge's construction, pre-construction, and operational phases will have low to moderately negative environmental effects. As the effective implementation and monitoring of the appropriate mitigation measures aid in reducing severe negative consequences, this study explored some impact mitigation strategies incorporating the principles of the mitigation hierarchy to avoid, minimize, restore, and offsets all the identified negative impacts of this project [24,45,46]. First, this study attempted to consider all potential alternate bridge locations that could aid in choosing an appropriate site and avoiding all adverse environmental effects (Fig. 3). For this purpose, we selected a total of 3 alternate bridge sites in addition to the proposed site. In this analysis, the location that was already proposed and located relatively close to the major road networks was deemed to be the most suited for the construction of the Boga Bridge and to have fewer negative environmental effects than other alternates [47].

We also thought option 1 as the best alternative after the proposed one for building the bridge because it would require less land to build the approach road, which will reduce negative environmental effects. On the other side, it will hamper the ferry movement for an extended period. Moreover, the depth of the river water at this point is much higher than any other location of this river due to frequent ferry movement and dredging. However, because of the river's meandering nature, option 2 was not practical. Additionally, this location was far from the main road, necessitating the acquisition of a lot of agricultural lands for the construction of the approach road and bridge, which will have more detrimental environmental effects. Similar to option 2, option 3 was deemed unsuitable because a new tiny bridge would need to be built across the little river it crossed, and the placement of the bridge was also far from the main road. The avoidance of negative impacts was not achievable because the proposed location was determined to be the best option for bridge construction. Therefore, this study furnished some effective environmental impact mitigation strategies to reduce all the adverse impacts as well as enhance positive impacts from specific project activities so that this bridge becomes a blessing instead of a curse in the future (Table 5).

**Table 5:** Proposed environmental impact mitigation measures.

Potential impacts	Proposed mitigation measures	References
Impact on topsoil and cropland	● Before being disposed of, contaminated soils should be treated using soil stabilization, soil washing, or chemical oxidation. ● Excavated top soils or other cut-off soils should be accumulated and preserved for reuse, especially clay soils to line up canals. ● Topsoil should be spread over to maintain the biological and physio-chemical activity of the soil. ● Afforestation and reforestation work at the construction site needs to be done right away after the work is finished.	[1,[48], [49], [50], [51]]
Loss of vegetation	● Planting trees on both sides of the approach road. ● Revegetation should be applied at the suitable locations after consulting with the local relevant authorities.	[8,41,[48], [49], [50], [51]]
Impact on agricultural land and loss of production	● Try to acquire the minimum amount of agricultural land possible. ● Reconstruction of damaged irrigation and drainage systems at a priority basis for sustainable agricultural production. ● Enhanced regional planning and the integration of land use and transportation planning can restore agricultural production. ● Non-agriculture and khash lands should bring under the agricultural practice for compensating reduced agricultural production.	[8,[48], [49], [50], [51], [52], [53]]
Resettlement and relocation	● Prioritize rehabilitation of any damaged utility services and personal property caused by the project activity ● Give the people who were relocated suitable land, money, and assistance so they can have options for sustainable livelihoods. ● Throughout each stage of the project, relocated individuals should be given preference for employment and working opportunities. ● Alternative livelihood options for the displaced small businessmen	[8,41,[49], [50], [51]]
Impact on water quality	● Controlling runoff, and deposing waste in the selected sites. ● Prevent the mixing of wastewater and other waste materials with river water. ● Before disposal, an effective Fenton-like technique should be used to remove contaminants from wastewater. ● Keep watch on water quality parameters such as turbidity, color, temperature, and suspended sediment concentrations to minimize water pollution. ● Before being dumped into the environment, sewage should be treated following national legal regulations.	[8,51,54,55]
Impact on air quality	● Construction materials such as excavated earth-dredged soil, sand, and gravel should be covered properly to minimize wind drift. ● Using machinery that is electrically powered rather than burning coal or oil. ● Regular monitoring of project site air quality. ● Excessively polluting construction equipment should be immediately banned from construction sites. ● Utilize less fuel when driving and keep construction equipment and vehicles in good working order by performing regular maintenance. ● At the construction site, all vehicles must move at a maximum speed of 30 km/h following BRTA laws and regulations to limit dust emissions. ● With the assistance of relevant authorities, new species of trees should be planted in the proper places.	[7,15,41,51,52]
Noise and vibration	● Priority should be made to reducing noise exposure by putting up noise barriers. ● Utilize machinery with the most recent noise-canceling technology. ● In vibration-sensitive regions, low-vibration generation equipment should be installed. ● Vehicles and equipment that make noise shouldn't be operating at night. ● Low-impact and alternative piling techniques, such as bore piles and micro piles, should be used instead of traditional impact hammer piling techniques. ● A proper schedule of noisy construction activities.	[7,8,15,52]
Waste generation and dumping	● Before beginning construction, estimate the volume of possible trash creation and create a strategy for disposing of it that works. ● Reduce the amount of sediment, organic matter, trash, debris, oil and grease, surplus nutrients, and other waste by using the 3R (Reduce, Recycle, and Reuse) strategy. ● Choose the best locations to temporarily store building site debris, then promptly remove it from these locations. ● Composting should be utilized to control organic waste and create organic fertilizer that farmers may use. ● For the treatment of wastewater, national standards should be used and followed.	[15,[49], [50], [51], [52],^56^]
Sedimentation	● Building barriers will lessen the amount of silt that reaches aquatic bodies. ● To reduce the flow of silt, spilled areas should be cleaned up. ● Construction sites can benefit from a buffer zone that is easily controlled by a significant quantity of sediment loss, such as one created by a turbidity curtain and control fences.	[57]
Changes in river flow	● Design appropriate river training work for maintaining actual river water flow. ● Any kind of waste should be dumped into the designated waste dumping area. ● To keep the river's actual flow intact, carry out planned dredging operations.	[15,57]
Impact on ecosystem health	● Before beginning construction, thoroughly study the wildlife or environment to identify any protected species and their habitats and to make recommendations for conservation measures to be taken during the construction process. ● Assure the ecosystem's components are still operating after construction work is finished. ● The introduction of an increasing number of tolerant species can restore affected ecosystems.	[[48], [49], [50], [51]]
Impact on endangered species	● Try to avoid endangered species habitats. ● Reduce noise levels at the construction site to lessen their impact on endangered species. ● Time constraints for construction activities can help recover endangered species. ● If there is no other option, create new habitats for endangered species.	[48,49,51,52]
Impact on wetland and fisheries	● Make sure there is enough room and a path for fish and other aquatic species to travel safely. ● Should provide a code of conducts to the general public and employees that prohibit fishing and hunting during and after construction. ● To restore the fisheries in impacted areas, fish screens should be installed and maintained.	[48,49,52]
Impact on flora and fauna diversity	● It is important to train construction workers to protect natural resources like flora and fauna. ● Should replant damaged and exposed areas with indigenous species from the area to encourage natural vegetation regrowth. ● Replanting of vegetation to create habitat for animals like birds and insects.	[7,49]
Impact on Char land	● The right use of fallow land can be a solution. ● Carefully select alignment and routes to reduce the impacts on char land ● The banks and buildings on char land can be adequately protected by morphological research and consideration.	[51,52]
Impact on Employment and lifestyle	● Employing locals for labor-related tasks while preserving gender equity. ● Based on the quality and requirements of the job, both male and female employees should eventually have access to a suitable pay structure and other social benefits. ● Generation of new sources of income and management diversify livelihood options for local people in the long run.	[8]
Accident and health risk	● Create and carefully abide by a traffic management strategy that includes warning signs and traffic guidance. ● It is important to implement road safety measures like speed bumps, safety signs, flagmen, loading zones, and parking spaces. ● Consider the location of the construction yard away from the local communities. ● Ensure the greatest level of worker safety during each stage of the construction process. ● Inform campers about PPE, sleeping arrangements, exterior doors, and a sufficient supply of clean water.	[7,41,49,50]

## 4 Discussion

All the development activities like bridge construction must have some potentially negative impacts on the surrounding environmental components though they are built to benefit humankind by making the communication system faster and easily assessable [3,4,15,37,41,57]. According to this study, the proposed Boga Bridge will have several negative impacts on the surrounding environment such as loss of agricultural land and production, loss of aquatic habitat, extinction of endangered species, degradation of water, air, and soil quality, a decline of ecosystem health, loss of flora & fauna diversity, change in river flow, and sedimentation, etc. during construction, maintenance and operation phases. Prior investigations carried out by Islam et al. and Hasan et al. during the beginning planning stage of this project also confirmed all these detrimental effects [1,8]. Additionally, the results of this study are consistent with recent EIA studies performed on the Khan Jahan Ali Bridge [4], Padma Multipurpose Bridge [5], Mymensingh Kewatkhali Bridge over the Old Brahmaputra River [49], Galachipa Bridge [7], Payra Bridge [14], Kashil Bridge over the Jhinai River at Tangail [58]. Furthermore, a recent study discovered that the Dharla Bridge's construction had several impacts on the hydrological and morphological features of the Dharla River [57].

On the other hand, this project's beneficial effects on road transportation, economic empowerment, industrialization, employment and lifestyle, gender promotion, growth of commercial facilities, landscape changes, and community upgradation were also noted in the study. These positive effects are consistent with those discovered during the project's initial planning stage by Islam et al. [1] and Hasan et al. [8]. Likewise, this study, EIA studies conducted on different bridge construction projects in Bangladesh such as Khan Jahan Ali Bridge [4], Padma Multipurpose Bridge [3], Mymensingh Kewatkhali Bridge over the Old Brahmaputra River [49], Galachipa Bridge [7], Payra Bridge [14], and Kashil Bridge over the Jhinai River at Tangail [15] also documented some positive effect on the economic condition, industrialization, employment and community up gradation and social status and people's lifestyle.

Our calculated environmental impact value "-2,” and evaluated project impact value estimated using Leopold matrix “-1.51” for this proposed project indicating that it might have a low negative consequence on the surrounding environment. However, environmental impact studies conducted for bridge construction projects in Bangladesh predicted both the positive and adverse environmental consequences and impact values based on the project extent and surrounding environments. For example, Islam et al. [14] estimated “+2” EIV for the Payra Bridge, and Islam et al. [15] calculated “+3” EIV for the Kashil Bridge over the Jhinai River at Tangail district. On the other hand, Islam et al. [1] estimated overall EIV at “-3” for the Boga Bridge project which is almost similar to our study. The tiny discrepancy might exist because Islam et al. completed their study almost 8 years ago, and the environment might have changed during that extended period. Additionally, a critical assessment of the project's environmental effects using the Leopold matrix predicted significant negative effects during construction, minor negative effects during pre-construction, and significant positive effects during operation, demonstrating the transient nature of the project's negative environmental effects.

Moreover, the analytical hierarchy process and multi-criteria decision analysis offered a comprehensive and in-depth look at how stakeholders feel about this project. In spite of some detrimental environmental consequences throughout its construction phase, this proposed project will help to make road transportation more convenient and easier as well as create opportunities for economic development in the long run. In addition, it is extremely important for the socioeconomic advancement of nearly 2 million residents of the southern upazilas of Bauphal, Dasmina, and Galachipa. That's why, the vast majority of respondents and stakeholders preferred to construct the bridge in the proposed location using the existing design after incorporating appropriate impact mitigation strategies. Similarly, the majority of prior impact assessment studies on bridge construction projects in Bangladesh encourage building bridges after applying appropriate and deliberate mitigating measures [1,[7], [8], [9],^14^,^15^,^49^]. Moreover, all potential negative environmental impacts identified in this study could be minimized by taking appropriate precautionary impact mitigation measures as this project has a low negative impact on the environment. Therefore, this study advises building the proposed Boga bridge after strictly implementing all of the impact mitigation strategies it offers, which incorporate the principles of the mitigation hierarchy. In addition to this, the responsible authority should develop a comprehensive monitoring, evaluation, and follow-up plan to make sure that these impact mitigation strategies are strictly followed from the start to end of the construction works to ensure the project's success and the best possible outcome [[59], [60], [61]]. On the other hand, the project's environmental effects will be much more severe than we had anticipated without the correct execution of any mitigation measures before beginning the project activity.

## 5 Limitation of the study

When assessing and evaluating potential environmental consequences, this study failed to consider the dynamics of climate change and its impact [62]. Additionally, while investigating mitigation strategies, neither climate change adaptation nor mitigation strategies were taken into consideration [41,63]. This study also failed to identify secondary and tertiary impacts this proposed project has as well as failed to differentiate among different short, medium, and long-term environmental consequences. Moreover, since Leopold matrix analysis is not mutually exclusive, there is a possibility of duplication when counting and assessing impacts [38]. The shortcomings of this study should all be addressed in future environmental impact assessment studies.

## 6 Conclusion

The southern coastal region of Bangladesh is receiving increased economic attention from the government and as a result, experiencing a revolutionary change in the infrastructure development. Since the completion of the Padma and Payra bridges, it has brought drastic changes in this region's economic empowerment and communication system. However, because they are still dependent on the old ferry system to cross the Lohalia River, the residents of Bauphal, Dashmina, and Galachipa upazilas are not getting actual benefits from the Padma and Payra bridges. Recently, the Bangladesh Road Transport Authority announced plans to build a bridge at Boga, Patuakhali, over the Lohalia River, which will finally alter their fate. This study was conducted to identify and evaluate all the beneficial and adverse impacts of this bridge construction project on the surrounding environmental components*.* According to this study proposed bridge infrastructure development has some adverse impacts on different ecological, physiochemical, socio-cultural, and human-interest parameters such as agricultural land and production loss, topsoil and vegetation damage, loss of aquatic habitat and endangered species, degradation of surface water and air quality, noise and soil pollution, impact on ecosystem health and change in the river route, etc. Both the environmental impact value (−2) and estimated project impact value using the Leopold matrix (−1.51) predicted an overall low negative environmental consequence for this proposed project activities. Although this project has certain adverse environmental effects during its many construction phases, this bridge will ultimately change the fate of coastal people's livelihood and lifestyle and bring rapid economic development through an easily assessable road transportation system after completion. This study found the extreme importance of the proposed Boga bridge for the socioeconomic advancement of this region as people will be freed from the extreme suffering of ferries and can easily travel directly to the capital Dhaka along with all southern districts. Moreover, most of the potential environmental consequences found in this study were temporary as they were only noticeable during different project construction activities, and in its operational stage, these adverse impacts will turn into great positive impacts. Additionally, all of these negative effects during the construction phase and any lingering effects after completion could be easily reduced by strictly enforcing the suggested precautionary impact mitigation measures in this study, which were developed incorporating the principles of the mitigation hierarchy. Finally, after weighing this project's massive importance and socioeconomic benefits as well as its all drawbacks and environmental consequences, this study concluded that the Boga Bridge over the Lohalia River should be built after strictly enforcing and monitoring all the impact mitigation strategies stated in this study to avoid, minimize, restore and offset all potential negative environmental effects of this project.

## Author contribution statement

Md. Tariqul Islam and Maksudul Islam: Conceived and designed the experiments; Performed the experiments; Analyzed and interpreted the data; Contributed materials, analysis tools or data; Wrote the paper.

Mawya Siddeqa: Conceived and performed the experiments; Analyzed and interpreted the data; Wrote the paper.

Ananya Mukherjee: Conceived and performed the experiments; Analyzed and interpreted the data; Wrote the paper.

Shakina Afroz Bithi: Performed the experiments; Wrote the paper.

Songita Mandal: Contributed materials, analysis tools or data.

## Data availability statement

Data will be made available on request.

## Declaration of competing interest

The authors declare that they have no known competing financial interests or personal relationships that could have appeared to influence the work reported in this paper.
