# Supplementary material for: An assessment of the potential environmental effects of bridge construction in Boga, Patuakhali, Bangladesh
Source: Heliyon. 2023 May 23;9(6):e16562. doi: 10.1016/j.heliyon.2023.e16562 (PMC10245150; doi:10.1016/j.heliyon.2023.e16562)
Supplement: Multimedia component 1 [file mmc1.docx]

**Survey Questionnaire**

**An Assessment of the Potential Environmental Effects of Bridge Construction Project in Boga, Patuakhali, Bangladesh**

[N.B. All of the information being collected under this module is only for research purpose at Patuakhali Science and Technology University]

| Form no: | Union Name: | Village Name: | Date: |
| --- | --- | --- | --- |

**A. Respondents profile:**

i. Name of the respondents:

ii. Age: iii. Sex: iv. Occupation: v. Cell no:

1. What is the current scenario of road transportation and communication with Barishal, Patuakhali and Dhaka in this area?

……………………………………………………………………………………………………………………………………………………………………………………………………

2. Recently government has decided to construct a bridge over the Lohalia river at Boga point. Do you think whether it is necessary to implement the proposed Boga Bridge project?

a) Yes b) No

If yes, how you and surrounding people will be benefited from the construction of this proposed bridge?

a.

b.

c.

3. Is there have any chance for the acquisition of your land for bridge (construction/rehabilitation/reconstruction)?

a) Yes b) No

If yes, then how much land will be required? What will be the impact of this acquisition upon you? …………………………………………………………………………………………………………………………………………………………………………………………………….

4. What type’s environmental impact may occur due to construction of Boga Bridge? (**Pre-construction Stage)**

a.

b.

c.

5. What type’s environmental impact may occur due to construction of Boga Bridge? (**Construction Stage)**

a.

b.

c.

6. What type’s environmental impact may occur due to construction of Boga Bridge? (**Post-construction Stage)**

a.

b.

c.

7. Is there have any impacts on minority or vulnerable groups (ex. Indigenous, tribal etc.)

a) Yes b) No

If yes, what are those impacts?

…………………………………………………………………………………………………………………………………………………………………………………………………….

8. Based on the current location, is the bridge is located in the best location?

a) Yes b) No

If no, then what’s the reason? Please specify

…………………………………………………………………………………………………………………………………………………………………………………………………….

9. What type of compensation the project authority has committed to you? Will it be enough to your losses?

a.

b.

c.

10. What are the strategies you consider will be useful to avoid, mitigate, restore and offsets all the environmental consequences of Boga Bridge construction project?

a.

b.

c.

*******Thanks for Your Valuable Time and Nice Cooperation!!******

| ……………………………………………  Signature of the interviewer (With date) | ……………………………………………  Signature of Interviewee (With Date) |
| --- | --- |

**Multi-criteria Decision Analysis (MCDA) and Analytical Hierarchy Process (AHP)**

Please define which criterion is more important, and how much more on a scale 1 to 9 where 1- Equal Importance, 3- Moderate importance, 5- Strong importance, 7- Very strong importance, 9- Extreme importance (2,4,6,8 values in-between).

**Table S1:** Checklist for the pairwise comparisons in the Analytical Hierarchy Process (AHP)

| **Number** | **Option A** | **Option B** | **Select A or B** | **Importance Scale**  **(Use scale 1-9)** |
| --- | --- | --- | --- | --- |
|  | Required no mitigation measures | Selection of alternative site |  |  |
|  | Required no mitigation measures | Modification of bridge design |  |  |
|  | Required no mitigation measures | Effective mitigation measures |  |  |
|  | Required no mitigation measures | Robust monitoring and evaluation |  |  |
|  | Required no mitigation measures | Early engagement of stakeholders |  |  |
|  | Required no mitigation measures | Capacitate and engage local community |  |  |
|  | Required no mitigation measures | Improve ferry facility |  |  |
|  | Required no mitigation measures | Bridge shouldn’t be constructed |  |  |
|  | Selection of alternative site | Modification of bridge design |  |  |
|  | Selection of alternative site | Effective mitigation measures |  |  |
|  | Selection of alternative site | Robust monitoring and evaluation |  |  |
|  | Selection of alternative site | Early engagement of stakeholders |  |  |
|  | Selection of alternative site | Capacitate and engage local community |  |  |
|  | Selection of alternative site | Improve ferry facility |  |  |
|  | Selection of alternative site | Bridge shouldn’t be constructed |  |  |
|  | Modification of bridge design | Effective mitigation measures |  |  |
|  | Modification of bridge design | Robust monitoring and evaluation |  |  |
|  | Modification of bridge design | Early engagement of stakeholders |  |  |
|  | Modification of bridge design | Capacitate and engage local community |  |  |
|  | Modification of bridge design | Improve ferry facility |  |  |
|  | Modification of bridge design | Bridge shouldn’t be constructed |  |  |
|  | Effective mitigation measures | Robust monitoring and evaluation |  |  |
|  | Effective mitigation measures | Early engagement of stakeholders |  |  |
|  | Effective mitigation measures | Capacitate and engage local community |  |  |
|  | Effective mitigation measures | Improve ferry facility |  |  |
|  | Effective mitigation measures | Bridge shouldn’t be constructed |  |  |
|  | Robust monitoring and evaluation | Early engagement of stakeholders |  |  |
|  | Robust monitoring and evaluation | Capacitate and engage local community |  |  |
|  | Robust monitoring and evaluation | Improve ferry facility |  |  |
|  | Robust monitoring and evaluation | Bridge shouldn’t be constructed |  |  |
|  | Early engagement of stakeholders | Capacitate and engage local community |  |  |
|  | Early engagement of stakeholders | Improve ferry facility |  |  |
|  | Early engagement of stakeholders | Bridge shouldn’t be constructed |  |  |
|  | Capacitate and engage local community | Improve ferry facility |  |  |
|  | Capacitate and engage local community | Bridge shouldn’t be constructed |  |  |
|  | Improve ferry facility | Bridge shouldn’t be constructed |  |  |
